# High rate of human enteroviruses among clinically suspected meningitis cases at selected Hospitals in Addis Ababa, Ethiopia

**DOI:** 10.1371/journal.pone.0258652

**Published:** 2021-11-11

**Authors:** Ashenafi Alemu Wami, Gadissa Bedada Hundie, Rozina Ambachew, Zenebe Gebreyohannes Berhe, Alem Abrha, Workeabeba Abebe, Dessalegen Abeje, Alene Geteneh, Adane Mihret, Andargachew Mulu

**Affiliations:** 1 Armauer Hansen Research Institute (AHRI), Addis Ababa, Ethiopia; 2 Department of Microbiology, Immunology, and Parasitology, St. Paul’s Hospital Millennium Medical College, Addis Ababa, Ethiopia; 3 School of Medicine, Addis Ababa University, Addis Ababa, Ethiopia; 4 Department of Medical Laboratory Science, College of Health Sciences, Woldia University, Woldia, Ethiopia; Institut Pasteur, FRANCE

## Abstract

**Background:**

Because of limited infrastructure and skilled human capital, the etiology of meningitis is rarely identified in developing countries like Ethiopia. This results in unnecessary antibiotics use, economic crisis, hospitalization, and related nosocomial infections. Thus, we aimed to assess the epidemiology of human enteroviruses (HEVs) among clinically suspected meningitis cases in Addis Ababa, Ethiopia.

**Method:**

A cross-sectional study was conducted from January to August 2020 at selected Hospitals in Addis Ababa, Ethiopia. Reverse transcriptase-polymerase chain reaction (RT-PCR) was conducted on cerebrospinal fluid (CSF) collected from 146 clinically suspected meningitis and bacterial culture-negative patients. SPSS v 21.0 was used for data analysis and bivariate correlation was done for the association between variables of interest.

**Results:**

HEVs were detected in 39 (26.7%) of the 146 clinically suspected meningitis cases. Most of the HEVs cases 28 (71.9%) were detected in younger-aged infants less than 1 year. The most commonly observed clinical manifestations were vomiting (75.5%) followed by fever **(**56.8%) and impaired consciousness or irritability (50.7%). The mean length of hospital stay for patients with enteroviral meningitis was 9 days. Many patients with HEVs were recovered with sequelae (46.2%), and HEVs has contributed for one out of the nine meningeal deaths (11.1%).

**Conclusions:**

HEVs were found to be the commonest cause of morbidity and mortality in all age groups. Many of the patients were mistreated with antibiotics and hospitalized. The detection of HEVs in 26.7% of clinically suspected meningitis cases indicated the need for molecular tests in investigating the etiology of meningitis. Therefore, we suggest the introduction of molecular tests as a routine practice in referral hospitals and the need to further characterize circulating HEVs strains.

## Introduction

Viruses have emerged as a significant cause of morbidity and mortality in all age groups under the era of declined incidence of bacterial meningitis [1, 2]. Human enteroviruses (HEVs) are the predominant cause of aseptic or viral meningitis globally. It accounts for more than half of all meningitis cases and affects all age groups, but infants and children are the most susceptible group [3–6]. Several studies conducted in different European and Asian countries estimated that 15% to 60% of viral meningitis cases were caused by non-polio human enteroviruses [7–10]. Unlike temperate region, in the tropical regions, HEVs associated with aseptic meningitis become incidents throughout the year without seasonal variation [11, 12].

In Africa, the poor resources and expense of diagnostic tests have constrained viral investigations. Hence, the exact disease burden and epidemiology of HEVs meningitis is not well known. Limited studies available thus far have assessed the extent of viral meningitis to vary between 8–65% of all meningitis cases [13–16]. Similarly, previous studies in Ethiopia estimated enteroviral meningitis to vary between 9% [17] and 12.8% [18]. This variation would be attributable to the spectrum of disease, seasonal distribution, age, immune status, and geographical locations of patients [19].

The overlapping clinical manifestations of meningitis caused by different etiologic agents, and the inability of clinical and routine laboratory investigation to distinguish bacterial from viral meningitis could result in misdiagnosis, mistreatment with antibiotics, and hospitalization. However, the advent of molecular techniques like RT-PCR has significantly improved the diagnosis of enteroviral meningitis, which subsequently reduces the use of antibiotics, decreases the length of stay in the hospital, and can help to prevent further spread of infection [20]. Early identification and differentiating viral from bacterial causes of meningitis is a critical step for improved management of patients. However, to the best of our knowledge, data regarding viral meningitis is lacking in Ethiopia. We, therefore, aimed to assess the epidemiology of HEVs meningitis among clinically suspected meningitis cases in selected hospitals.

## Materials and methods

### Study setting

A cross-sectional study was conducted from January-August 2020 in five public Hospitals in Addis Ababa, Ethiopia: All Africa Leprosy Rehabilitation and Training Hospital (ALERT), Tikur Anbessa Specialized Hospital (TASH), Yekatit 12 Hospital, St. Peter’s Specialized Hospital, and St. Paul’s Hospital Millennium Medical College (SPHMMC). All of the institutions are fully-fledged government-affiliated hospitals serving as referral hospitals for people who come from all over the country. The target populations of this study were suspected patients of all age and sex in the five public teaching and referral hospitals during the study period.

### Inclusion and exclusion criteria

Aseptic/viral meningitis is a disease with acute onset of symptoms, obvious signs of meningeal involvement, and no growth on routine bacterial culture [21–23]. This study included patients who were clinically suspected of meningitis, negative for routine bacterial culture, and willing to participate in the study. Patients positive for routine bacterial culture were excluded.

### Specimen collection

Cerebrospinal fluid was collected from 146 patients clinically suspected meningitis cases based on routine clinical practice, either at admission or later during the hospital stay. Routine diagnostic tests were conducted on all of the collected CSF samples. Leftover CSF samples were stored at -20°C and transported by dry ice to the Armauer Hansen Research Institute (AHRI) for further investigation. The demographic and clinical data such as date of birth, gender, onset of illness, clinical presentation, laboratory findings of the blood and CSF tests, antimicrobial treatment administered, length of hospital stay and clinical outcomes were collected by physician and trained nurses.

### Nucleic acid extraction and RT-PCR

Viral nucleic acid was extracted from 200 μl of CSF samples using DAAN Gene (Da An Gene Co., Ltd, of Sun Yat-Sen University, China) following the manufacturer’s instructions. Reverse transcription was conducted using HEVs specific reverse primer: PanEV2-5ʹNTR R: 5ʹ-CACCCAAAGTAGTC GGTTCCGC-3ʹ [24] in a 20 μL reaction mixture containing 6μl of RNase free water, 1μl of 5mM dNTP, 1 μl of 2uM EV reverse primer, 5μl RNA template, 4μl of 10x SSV buffer, 1μl 10unit/μl RNase inhibitor, 1μl of 100mM DTT and 1μl of superscript IV reverse transcriptase (Invitrogen, USA). The mixture was incubated at 50 ^0^C for 10 minutes to synthesis cDNA, and the reaction was inactivated by incubating at 80°C for 10 minutes. The cDNA product was then amplified using conventional PCR consisting of 10 μl of hot star master mix (Qiagen, Hilden, Germany), 0.5 μl each of forward (PanEV2-5ʹNTR F: 5’CAT GGT GCG AAG AGT CGA TTG A3’) and reverse (PanEV2-5ʹNTR R) primer: 4μl of molecular grade water, and 5μl cDNA template; with final reaction volume of 20μl to get an amplicon size of 144bp. The amplification was done for initial denaturation at 95°C for 15 minutes, then 40 cycles of denaturation at 95°C for 15 seconds, followed by annealing at 60°C for 60 seconds, and extension at 60°C for 60 seconds. PCR products were visualized on a 2% agarose gel. This protocol was modified from a recently published article [18].

### Quality control

The quality of data was ensured through pretesting of the data collection sheet, proper training of data collectors, and sticking to SOPs of the AHRI molecular laboratory. NATrol™ EV (Enterovirus) (Helvetica Health Care, Geneva, Switzerland) and RNase/DNase free water was used as positive and negative test control, respectively.

### Statistical analysis

Data entry and analysis were done using SPSS version 21.0 statistical software. Binary logistic regression was conducted for statistical associations between variables. A *p-value* < 0.05 was considered statistically significant.

### Ethical issues

The study protocol has been approved by the Institutional Review Board (IRB) of St. Paul’s Hospital Millennium Medical College (SPHMMC) (Pm23/423), and by the collaborative Ethical Review Committee of Armauer Hanson Research Institute (AHRI) and All Africa Leprosy Rehabilitation and Training Hospital (ALERT) (AAERC) (PO/04/20). Support letter/ official permission letter was obtained from each health facility. A written informed consent/assent was obtained from study participants, and parents or guardians of children, before including them in the study. The study participants right to refuse or not give CSF samples without affecting their routine medical services were also granted. Samples were coded to keep the confidentiality of the study participants’ personal information.

## Result

### Socio-demographic and clinical characteristics

A total of 146 clinically suspected meningitis cases with negative bacterial cultures were enrolled in this study. The male to female proportion was 1.2:1, which indicates that cases were distributed nearly equally regardless of gender. The mean (± standard deviation) age of the study participants was 7±14 years, ranging from one day to 74 years. More than two-thirds of patients were under one year old, including 39.0% neonates (age < 28 days), and 28.1% infants aged between 29 days to one year (Table 1). Overall, HEVs were detected in 39 (26.7%) of the total 146 study participants who were clinically suspected of meningitis and negative for routine bacterial culture. HEVs were detected mostly (67.1%) from infants aged less than one year.

**Table 1:** Bivariate analysis of socio-demographic and clinical characteristics of patients with HEVs positivity at selected hospitals in Addis Ababa, Ethiopia.

Characteristics (n = 146)		Freq. N (%)	HEVs+ No (%)	COR (95%CI)	*P- value*
Sex	Female	66(45.2%)	20(51.3%)	1.396(0.669,2.912)	0.374
	Male	80(54.8%)	19(48.7%)	1	
Age (years)	<28 days	57(39.0%)	16(41.0%)	2.927(0.600,14.274)	0.184
	30days-1 year	41(28.1%)	12(30.8%)	3.103(0.613,15.706)	0.171
	1–5	13(9%)	5(12.9%)	4.687(0.736,29.834)	0.102
	6–14	14(9.6%)	2(5.1%)	1.250(0.153,10.226)	0.835
	15–18	4(2.7%)	2(5.1%)	7.500(0.645,87.193)	0.107
	>18	17(11.6%)	2(5.1%)	1	
Antibiotics use before LP	No	82(56.2%)	26(66.7%)	1	
	Yes	64(43.8%)	13(33.3%)	0.549(0.255,1.181)	0.125
Fever	No	63(43.2%)	18(46.2%)	1	
	Yes	83(56.8%)	21(53.8%)	0.847(0.405,1.770)	0.658
Headache	No	105(71.9%)	29(74.4%)	1	
	Yes	41(28.1%)	10(25.6%)	0.845(0.368,1.941)	0.692
Impaired consciousness	No	72 (49.3%)	16(41%)	1	
	Yes	74 (50.7%)	23(59.0%)	1.578(0.751,3.316)	0.228
Vomiting	No	36 (24.7%)	6(15.4%)	1	
	Yes	110 (75.3%)	33(84.6%)	2.143(0.815,5.634)	0.122
Stiffness	No	109 (74.7%)	27(69.2%)	1	
	Yes	37 (25.3%)	12(31.8%)	1.458(0.646,3.291)	0.364
Photophobia	No	120 (82.2%)	31(79.5%)	1	
	Yes	26 (17.8%)	8(20.5%)	1.276(0.505,3.227)	0.607
Seizure attack	No	112 (76.7%)	30(77.0%)	1	
	Yes	34 (23.3%)	9(23.0%)	1.038(0.434,2.483)	0.934
Dizziness	No	119(81.5%)	28(71.8%)	1	
	Yes	27 (19.5%)	11(28.2%)	2.234(0.930,5.369)	0.072
Rash	No	137 (93.8%)	36(92.3%)	1	
	Yes	9 (6.2%)	3(7.7%)	1.403(0.333,5.904)	0.644
Outcome	Dead	9(6.2)	1(2.5%)	0.625(0.048,8.201)	0.720
	Discharged	29(19.9)	6(15.4%)	1.304(0.223,7.613)	0.768
	Fully recovered	48(32.9)	12(30.8%)	1.667(0.319,8.703)	0.545
	Recovered with sequelae*	48(32.9)	18(46.2%)	3.000(0.590,15.262)	0.186
	Transferred	12(8.2)	2(5.1%)	1	
Hospital stays	<5days	29 (19.9%)	1(2.5%)	1.080(0.292,3.989)	.908
	6–10 days	39 (26.7%)	6(15.4%)	1.200(0.348,4.137)	.773
	11-15days	47 (32.2%)	12(30.8%)	0.568(0.159,2.027)	.384
	16-20days	14 (9.6%)	18(46.2%)	0.655(0.126,3.404)	.614
	>21days	17 (11.6%)	2(5.1%)	1	

Fever, temperature ≥ 38°C; COR, crude odds ratio; CI, confidence interval; Abcs, Antibiotics; LP, lumbar puncture.

*Meningitis related complications including hearing loss, cognitive impairment, and recurrent seizures.

Vomiting or reduced ability to suck 33 (84.6%), impaired consciousness 23 (59.0%), and fever 21 (53.8%) were the most common clinical presentations among positive cases at admission to the hospitals. Many of the patients had a history of prior antibiotics use before the spinal tap and were also hospitalized for 5–15 days. According to this finding, 39 (26.7%) of patients were mistreated with antibiotics for bacteria where the actual etiologies were HEVs. Regarding the clinical outcome of patients with HEVs infection; more than 46% of patients recovered with sequelae, followed by full recovery (30.8%). A total of 9 patients died of meningitis, of which 1 death was attributable to HEVs. Interestingly, none of the demographics, clinical, and laboratory findings has shown an association with HEVs positivity (***p>0*.*05***) (Table 1).

### Laboratory findings of study participants

The white blood cells (WBC) count for newborns 7640 to 22160 cells/mm^3^, children 5000 to 14,000/ mm^3^, and children over 7 years and adults 3500–10,500 cells/mm^3^ was considered as a normal reference range [25–28]. In this study, WBC was performed for about 88% (n = 128) of the study participants. The mean WBC count of study participants was 12980 cells/mm^3^, ranging from 1800 to 66,500 cells/mm^3^. Relatively a higher WBC count of 20 (51.3%) was observed among HEVs positive cases. Among HEVs positive cases, the majority 30 (77.0%) of CSF samples appeared clear. Astonishingly, none of the laboratory finding was statistically correlated with HEVs meningitis (S1 Table).

## Discussion

Human enteroviruses remain the most common causes of aseptic meningitis [13, 29] varying between 6% and 64% worldwide [30]. However, viral etiologies of meningitis are rarely identified in developing countries like Ethiopia owing to a lack of advanced laboratory settings. In this study, we investigate enteroviral meningitis in five public teaching hospitals using molecular diagnostic techniques. We found that 39 (26.7%) of the clinically suspected meningitis cases are due to HEVs infection.

Although the distribution of HEVs meningitis is likely to vary between age groups, our finding was consistent with the finding in Kuwait (24%) [31] and old study in Belgium (27.1%) [7]. However, compare to the findings in Athens (48.9%) [32], France (43.4%) [33], Ireland (61.9%) [23], South Korea (38.4%) [8], Iran (65%) [34] and Egypt (56%) [13], the prevalence in the present study was relatively lower. Nevertheless, the burden of HEVs meningitis in this study was higher compared to findings in USA (12.1%) [35], Cyprus (11.1%) [11], Brazil (15.8%) [36], China (19.2%) [37], and Palestine (18.5%) [30]. This discrepancy could be explained by the difference in age of patients, population location, and seasonal distribution of cases [19, 30, 31, 34].

In Africa, the incidence of aseptic or viral meningitis was shown to vary between 8% in Malawi [15] and 56% in Egypt [13]. The current finding also enlightens this fact. But our result was higher compared to some of the studies in African settings: Uganda (5.9%) [38], Tunisia (9.8%) [16] and South Africa (17%) [9] and to the previous studies in Ethiopia, 9% [17] and 12.8% [18]. The difference might be due to variation in clinical diagnosis, geography, type of study participants, sample size, age, molecular methods used in extraction or amplification [17, 35, 37].

Human enteroviruses remain the commonest causes of childhood meningitis globally [39, 40], and being a young age was reported as an important contributing factor for having viral meningitis [40]. Our study showed that majority of infants (72%) aged less than one year were more susceptible to HEVs meningitis, although it’s statistically insignificant. This finding was in agreement with studies in Iran [41], Palestine [30], China [37], and Ethiopia [17, 18], which showed that younger aged patients were more affected with enteroviral meningitis. The possible justification for this finding could be the less developed immune system in neonates and infants, which makes them more susceptible to enteroviral infections [30].

It is well noted that sex can be an important biological variable in the immune response to infectious diseases. In the present study, the significance of sex with enteroviral meningitis was assessed. Although the association was statistically insignificant (*P > 0*.*05*), more than half (51.3%) of positive cases were females regardless of the higher proportion of males. This result was contradictory to the study found in Palestine, where about two-thirds of positive cases were males [30].

Diagnosis of meningitis based on clinical criteria is very challenging in distinguishing viral from bacterial meningitis due to the overlapping symptoms [6, 20]. This made 26.7% of our patients be treated with antibiotics inappropriately instead of giving supportive care. In the present study, vomiting (75.3%), fever (57.5%), and impaired consciousness (50.7%) were the predominant clinical manifestations. This is in agreement with the study in Greece [32], China [37], South Korea [8], and Egypt [13] where fever, headache, and vomiting were the most common clinical symptoms in the majority of patients.

For a definitive diagnosis of meningitis, analysis of CSF is useful in distinguishing some of the etiologic organisms. In the current study, 77% of CSF appeared clear as expected in viral meningitis [42]. However, turbidity does not guarantee the absence of viral etiologies. In this study, 15.3% of HEVs were recovered in turbid CSF, and CSF pleocytosis was noted only in 12.9% of confirmed HEVs cases. Such findings were in line with other studies from South Korea [43], Palestine [30], Canada [44], and Iran [41]. Regarding the outcome of HEVs positive patients, many were recovered with sequelae (46.2%), with only one death registered. The proportion of death possibly attributed to HEVs was 11.1% (1/9). The difference in demographic, clinical, and laboratory findings was not statistically significant in our study (p > 0.05) similar to study elsewhere [13].

To our knowledge, this study is the first to report molecular methods for meningitis diagnosis by involving multicenter health institution in Ethiopia. The high rate of HEVs detection among clinically suspected meningitis cases indicated that the worth of molecular tests in identifying viral etiologies of meningitis. Therefore, we suggest the application of molecular tests as a routine practice in referral hospitals in Ethiopia and also the need of further study to the circulating HEVs. We also recommended further surveillance study to investigate the burden of HEVs at the national level.

### Limitation of the study

This study was limited to HEVs and missed other important viral etiologies of meningitis. So that we were unable to determine the proportions HEVs relative to other pathogens.

## Supporting information

S1 Table Association of laboratory findings with HEV positivity at selected hospitals in Addis Ababa, Ethiopia. (DOCX)
