# Supplementary material for: High rate of human enteroviruses among clinically suspected meningitis cases at selected Hospitals in Addis Ababa, Ethiopia
Source: PLoS One. 2021 Nov 11;16(11):e0258652. doi: 10.1371/journal.pone.0258652 (PMC8584720; doi:10.1371/journal.pone.0258652)
Supplement: S1 Table — (DOCX) [file pone.0258652.s001.docx]

**S1 Table: Association of laboratory findings with HEV positivity** at selected hospitals in Addis Ababa, Ethiopia.

| Characteristics |  | Freq. No (%) | HEVs + No (%) | COR (95%CI) | P- value |
| --- | --- | --- | --- | --- | --- |
| CSF appearance | Bloody | 13(8.8%) | 3(7.7%) | 0.860(0.222,3.336) | 0.827 |
|  | Yellowish | 1(0.7%) | 0(00.0%) | .000 |  |
|  | Turbid | 16(11.0%) | 6(15.3%) | 1.720(0.576,5.137) | 0.331 |
|  | Clear | 116(79.5%) | 30(77.0%) | 1 |  |
| WBC | Below normal | 5(3.4%) | 0(00.0%) | .000 |  |
|  | Higher | 70(47.9%) | 20(51.3%) | 1.067(0.485,2346) | 0.591 |
|  | Normal | 53(36.3%) | 15(38.5%) | 1 |  |
| Blood neutrophil count | Below normal | 27(18.5%) | 7(17.9%) | 1.009(0.363,2.804) | 0.987 |
|  | Higher | 34(23.3%) | 11(28.2%) | 1.379(0.557,3.411) | 0.487 |
|  | Normal | 65(44.5%) | 17(43.6%) | 1 |  |
| Blood lymphocyte count | Below normal | 49(33.6%) | 15(38.5%) | 1.018(0.418,2.480) | 0.969 |
|  | Higher | 36(24.7%) | 7(18.0%) | 0.557(0.195,1.594) | 0.275 |
|  | Normal | 43(29.5%) | 13(33.3%) | 1 |  |
| CSF protein level | Below normal | 6(4.1%) | 0(00.0%) | .000 |  |
|  | Higher | 43(29.5%) | 11(28.2%) | 3.781(0.437,32.74) | 0.227 |
|  | Normal | 12(8.2%) | 1(2.6%) | 1 |  |
| CSF glucose level | Below normal | 9(6.2%) | 4(10.3%) | 3.400(0.741,15.60) | 0.115 |
|  | Higher | 11(7.5%) | 0(00.0%) | .000 |  |
|  | Normal | 42(28.8%) | 8(20.5%) | 1 |  |
| CSF cell count | Higher | 16(11.0%) | 5(12.9%) | 1.189(0.346,4.089) | 0.784 |
|  | Normal | 47(32.2%) | 13(33.3%) | 1 |  |
| CSF lymphocyte count | Higher | 16(11.0%) | 5(12.8%) | 3.636(0.353,37.45) | 0.278 |
|  | Normal | 9(6.2%) | 1(2.6%) | 1 |  |
| CSF polymorphs count | Higher | 16(11.0%) | 5(12.8%) | 3.636(0.353,37.45) | 0.278 |
|  | Normal | 9(6.2%) | 1(2.6%) | 1 |  |

COR, crude odds ratio; CI, confidence interval
